# Value of Rectal Alimentation

**Published:** 1915-11

**Authors:** 


**Value of Rectal Alimentation.** Dr. Harry Adler, Baltimore. The usual method of feeding is unsatisfactory. The drop (Murphy) method, with peptonized milk and sugar solution, is markedly less annoying. In our experiments, the largest amount of nitrogen that we were able to supply was 8.9 gm., of which 8.06 gm. was returned with the stools. According to the observation of Dr. Muller, the stools of the individuals taking absolutely no nourishment contain 0.2 gm. per diem. With this deducted from our estimations of the returned nitrogen, we have in no case observed an absorption of more than 50 per cent. of the protein supplied; and it has fallen to as low as 30 per cent. The average amount of nitrogen absorbed in these cases was 1.14 gm. per diem. When we compare the amount of nitrogen lost by these cases with the amount that it was possible for them to absorb by colonic feeding, we realize how little we can accomplish. The difference between nutritional enteroclysis and normal salt enteroclysis is little over one-tenth of the tissue albumin loss per diem; over the periods of time that rectal feeding is usually carried out, it is, from a practical standpoint, an almost negligible quantity.

Discussion.

Dr. Jacob Kaufmann, of New York: Some of the nitrogen found in the stools is derived from the secretions, and this constitutes a possible source of error in making the estimations. I do not think that there is absolute proof of the uselessness of nutrient enemas.

Dr. Max Einhorn, of New York: We should try to give glucose by rectum, as it is the best material.

Dr. Franklin W. White, of Boston: I think it is valuable to contrast with this piece of work done by Dr. Adler the fact that nitrogenous equilibrium can be maintained by duodenal feeding. You can get it all in by the duodenum, but not by the rectum.

Dr. Louis M. Gompertz, of New Haven, Conn.: In some experiments that we conducted, a few years ago, it was found that the rectum is capable of salt absorption to about the same degree as the stomach. Experiments were also made with various other solutions. While I believe rectal feeding to be a pure makeshift, half a loaf is better than none. The use of salt solutions, water and dextrose solutions will tide the patients over the critical period required for rectal feeding.

Dr. John C. Hemmeter, of Baltimore: I should like to know whether the calculation that you can supply only one-tenth of nitrogen required by colonic feeding was based on the metabolism experiments made during rest or during activity. During rest, the food taken is entirely used in keeping up the heat of the body, which can be done without food, by artificial means.

Dr. Harry Adler, of Baltimore: The patients were absolutely at rest, in bed, or they could not have been fed by the drop method. We could supply only one-tenth of the amount needed when at rest. It is impossible to estimate accurately the absorption of sugar. We cannot give large amounts of milk by the drop method, because the rectum soon becomes irritated. We gave 8 ounces at a feeding, and gave three feedings a day. (Report of the Am. Gastro-enterologic Ass'n. in Practologist, Sept.)

